# Strukturierte Ersteinschätzung in der Notaufnahme mittels des intelligenten Assistenzdienstes OPTINOFA: Ergebnisse einer multizentrischen, clusterrandomisierten und kontrollierten Interventionsstudie in Deutschland

**DOI:** 10.1007/s00063-024-01229-6

**Published:** 2024-12-16

**Authors:** Sabine Blaschke, Harald Dormann, Rajan Somasundaram, Christoph Dodt, Ingo Graeff, Hans-Jörg Busch, Bernadett Erdmann, Marc Wieckenberg, Christoph Haedicke, Katrin Esslinger, Elisabeth Nyoungui, Tim Friede, Felix Walcher, Julia Talamo, Julia K. Wolff, OPTINOFA Study Group, Wilhelm Behringer, Ulrich Heida, Thomas Ruhnke, Christian Günster, Patrik Dröge, Michael Schmucker, Martin Haag, Michael Dietrich, Wiebke Schirrmeister, Felix Greiner, Paul Ludolph, Hans-Dieter Nolting, Kerstin Pischek-Koch, Stefanie Wache, Irina Chaplinskaya-Sobol, Dagmar Krefting, Kai Antweiler, Eva Hummers, Marina Karg, Jennifer Lenz, Kathrein Munski, Andreas Brockmann, Wiebke Boehne, Heike Teupe

**Affiliations:** 1 Zentrale Notaufnahme, Universitätsmedizin Göttingen (UMG), Robert-Koch-Str. 40, 37075 Göttingen, Deutschland; 2 Zentrale Notaufnahme, Klinikum Fürth, Fürth, Deutschland; 3 Zentrale Notaufnahme und Aufnahmestation, Campus Benjamin Franklin, Charité – Universitätsmedizin Berlin, Berlin, Deutschland; 4 Klinik für Akut- und Notfallmedizin, München Klinik Bogenhausen, München, Deutschland; 5 Abteilung für Klinische Akut- und Notfallmedizin, Universitätsklinik Bonn, Bonn, Deutschland; 6 Zentrum für Notfall- u. Rettungsmedizin, Universitäts-Notfallzentrum, Universitätsklinikum Freiburg, Freiburg, Deutschland; 7 Zentrale Notaufnahme, Klinikum Wolfsburg, Wolfsburg, Deutschland; 8 Zentrale Notaufnahme, Evangelisches Krankenhaus Göttingen-Weende, Göttingen, Deutschland; 9 Zentrale Notaufnahme, Städtisches Klinikum Braunschweig, Braunschweig, Deutschland; 10 Institut für Medizinische Statistik, Universitätsmedizin Göttingen (UMG), Göttingen, Deutschland; 11 AKTIN-Notaufnahmeregister, Universitätsklinik für Unfallchirurgie, Otto-von-Guericke-Universität Magdeburg, Magdeburg, Deutschland; 12 IGES-Institut, Berlin, Deutschland; 13 Institut für Community Medicine, Abteilung für Sozialmedizin und Prävention, Universitätsmedizin Greifswald, Greifswald, Deutschland

**Keywords:** Zentrale Notaufnahme, Klinische Notfall- und Akutmedizin, Triage, Strukturierte Ersteinschätzung, Intelligenter Assistenzdienst, Digitales Ersteinschätzungsinstrument, Qualitätsindikatoren, Emergency department, Clinical emergency and acute medicine, Triage, Structured assessment, Intelligent assistant service, Digital assessment instrument, Quality indicators

## Abstract

Die Organisation der Notfallversorgung in Deutschland bedarf einer grundlegenden Reform. Die Steuerung der Patientenströme in den ambulanten bzw. stationären Sektor stellt hierbei eine der zentralen Herausforderungen dar.

Im Innovationsfondsprojekt OPTINOFA wurde ein neues Triageinstrument zur strukturierten Ersteinschätzung von Behandlungsdringlichkeit und Notfallversorgungsstufe entwickelt und in einer clusterrandomisierten kontrollierten Studie im Stepped-wedge-Design in 8 Modellkliniken (davon 2 Per-protocol-Modellkliniken) im Zeitraum vom 01.07.2019 bis zum 31.05.2021 evaluiert. Zudem wurden Vergleichsdaten für allgemeine zeitliche Trends von einer Modellklinik im gleichen Zeitraum erhoben. Der primäre Endpunkt der Studie umfasste den Anstieg der Weiterleitung in den ambulanten Sektor; die sekundären Endpunkte bezogen sich auf Outcome, Prozess- und Qualitätsindikatoren sowie Kosten der Notfallbehandlung.

Im Kontrollzeitraum wurden in den Modellkliniken *n* = 46.558 und im Interventionszeitraum *n* = 37.485 Notfälle in die Studie eingeschlossen. Für den primären Endpunkt ergab sich in den Per-protocol-Modellkliniken ein signifikanter Anstieg der Zuweisungen in den ambulanten Sektor (*p* < 0,001; OR = 10,59). In Bezug auf die Wartezeiten fand sich eine signifikante Reduktion um durchschnittlich 20 min im Interventionszeitraum. Die stationäre Aufnahmerate innerhalb von 3 Tagen nach primär ambulanter Notfallbehandlung blieb im Interventionszeitraum stabil. In der Kostenanalyse ergab sich keine Kostensteigerung innerhalb von 28 Tagen nach der Notaufnahmevorstellung.

Im Innovationsfondsprojekt OPTINOFA konnte ein valider Assistenzdienst zur strukturierten Ersteinschätzung von Behandlungsdringlichkeit und Versorgungsstufe in der Notaufnahme entwickelt und als digitales Triageinstrument im interoperablen Format zur Verfügung gestellt werden. Die Studienergebnisse belegen ein großes Potenzial für die OPTINOFA-Triage zur transsektoralen Steuerung der Patientenströme in der Notfall- und Akutmedizin.

**Zusatzmaterial online:**

Zusätzliche Informationen sind in der Online-Version dieses Artikels (10.1007/s00063-024-01229-6) enthalten.

## Abstract (en)

In Germany, a substantial reform of emergency care is strictly recommended. Regulation of patient flows into the ambulatory and stationary sectors remains a major issue.

In the OPTINOFA project funded by Innovationsfunds, a new triage system was developed for a structured primary evaluation of both urgency and care level of emergency cases. OPTINOFA was evaluated in a cluster-randomized, controlled multicenter trial using a stepped-wedge design in eight emergency departments (ED) from 1 July 2019 to 31 May 2021. Additionally, data from one ED were used for comparison of temporal changes without intervention. The primary study endpoint represented the increase of patient transfers to the ambulatory sector; secondary endpoints included the outcome, process and quality indicators as well as mean emergency care costs.

In the study, 46,558 emergency cases were included in the control period and 37,485 emergency cases in the intervention period. Concerning the primary endpoint, a significant increase of transfers to the ambulatory sector were detected in the per-protocol EDs (*p* < 0.001, odds ratio = 10.59). Waiting times were significantly reduced by an average 20 min in the intervention phase. Furthermore, a stable admission rate was found within 3 days after initial ED presentation. Cost analysis revealed no increase of treatment expenses within 28 days after ED admission.

In this project a valid assistant service for structured primary evaluation of urgency and care level was successfully developed for emergency cases and served as a digital triage instrument with interoperable format. Clinical trial results revealed great potential for the OPTINOFA triage system to control patient flows in emergency and acute medicine.

In Deutschland ist die Notfallversorgung derzeit u. a. aufgrund der fehlenden Steuerung der Patientenströme in den Zentralen Notaufnahmen nicht bedarfsgerecht. Zur Lösung dieser Problematik wurde im Innovationsfondsprojekt OPTINOFA ein neues, digitales Triageinstrument für die strukturierte Ersteinschätzung von Behandlungsdringlichkeit und Versorgungsstufe in der Notaufnahme entwickelt und in einer prospektiven, multizentrischen, clusterrandomisierten und kontrollierten Interventionsstudie evaluiert.

## Hintergrund und Fragestellung

In Deutschland verzeichneten die Zentralen Notaufnahmen (ZNA) der Krankenhäuser seit Beginn der letzten Dekade einen deutlichen Fallzahlanstieg [5]. Nach passagerer Reduktion in der Coronapandemie liegen die jährlichen Fallzahlen stabil bei ca. 22,6 Mio. Notfällen. Diese hohe Inanspruchnahme der Notaufnahmen bei begrenzten räumlichen und personellen Ressourcen führt zu rezidivierenden Overcrowding-Szenarien [27]. Hierdurch resultieren Verzögerungen in der Notfallversorgung, lange Wartezeiten und Verweildauern und damit ein Anstieg der Risiken der Notfallbehandlung bis hin zur Erhöhung der Mortalitätsrate [1]. Darüber hinaus entsteht ein finanzielles Defizit insbesondere für ambulante Notfallbehandlungen [12].

Ursachen für das hohe Patientenaufkommen stellt neben der demografischen Entwicklung und zunehmenden Multimorbidität die inadäquate Inanspruchnahme der Notaufnahmen dar [19]. Bedingt durch die fehlende Steuerung der Patientenströme hat dabei der Anteil ambulanter Notfallbehandlungen überproportional zugenommen [12, 20]. Veränderungen in den vertragsärztlichen Versorgungsstrukturen [16], patientenseitig steigende Qualitätsansprüche, Öffnungszeiten 24/7 und die Substitution der fachärztlichen Versorgung tragen hierzu wesentlich bei [21, 26].

Eine ambulante Notaufnahmebehandlung erfolgt derzeit gemäß Gutachten der Deutschen Krankenhausgesellschaft (DKG) in etwa 60 % der Fälle; davon könnten ca. ein Drittel auch im vertragsärztlichen Bereich versorgt werden [12]. Daher wird übereinstimmend von den Fachgesellschaften Deutsche Gesellschaft für Interdisziplinäre Notfall- und Akutmedizin (DGINA) und Deutsche Interdisziplinäre Vereinigung für Intensivmedizin und Notfallmedizin (DIVI), der DKG, dem Spitzenverband der gesetzlichen Krankenversicherungen (GKV) und der kassenärztlichen Bundesvereinigung (KBV) eine sektorenübergreifende Reform der Notfallversorgung gefordert [8, 18, 24, 25]. Mit dem Krankenhausstrukturgesetz [9] wurde die Zusammenarbeit zwischen Notaufnahmen und vertragsärztlichem Notdienst gesetzlich vorgegeben (§ 75 Abs. 1b SGB V). Das Bundesministerium für Gesundheit hat im Jahr 2020 einen Referentenentwurf für die Reform der Notfallversorgung vorgelegt [6]. Im Rahmen des Gesundheitsversorgungsweiterentwicklungsgesetzes [10] wurde zudem festgelegt, dass der Gemeinsame Bundesausschuss (G-BA) Vorgaben zu einer strukturierten Ersteinschätzung des notfallmedizinischen Versorgungsbedarfs von Personen, die sich zur Notfallbehandlung an ein Krankenhaus wenden, beschließen soll (§ 120 Abs. 3b SGB V). Die Entwürfe hierzu [14] sowie die vierte Stellungnahme der Regierungskommission [5] zur Reform der Akut- und Notfallversorgung liegen vor.

Zur Steuerung der Patientenströme in die Versorgungsebenen „Notaufnahme im Krankenhaus“ (stationäre Notfallversorgung) und „niedergelassene Praxis“ (ambulante Notfallversorgung) gibt es jedoch in Deutschland bis dato kein validiertes System: Die Ersteinschätzung der Behandlungsdringlichkeit, sog. Triage, erfolgt in deutschen Notaufnahmen überwiegend mit den Triagesystemen Emergency Severity Index (ESI) oder Manchester Triage System (MTS; [7, 29]). Diese Systeme weisen eine unterschiedliche Performance auf und kategorisieren nur die Behandlungsdringlichkeit [28, 30], nicht jedoch die erforderliche Versorgungsstufe.

Im Rahmen des vom Innovationsausschuss (G-BA) geförderten Projekts „Optimierung der Notfallversorgung durch strukturierte Ersteinschätzung mittels intelligenter Assistenzdienste“ (Akronym: OPTINOFA; FKZ 01NVF 17035) wurde dazu ein intelligenter Assistenzdienst entwickelt, der eine strukturierte Ersteinschätzung von Behandlungsdringlichkeit *und* gleichzeitig der Versorgungsstufe von Notfällen erlaubt und damit erstmalig eine sektorenspezifische Zuweisung ermöglicht [15]. Die strukturierte Ersteinschätzung mittels OPTINOFA wurde nachfolgend in einer clusterrandomisierten, kontrollierten Studie im Stepped-wedge-Design mit externer Kontrollbedingung unter Beteiligung von 11 Modellkliniken bundesweit evaluiert. Das Design wurde gewählt, um die Rahmenbedingungen in Kontroll- und Interventionsphase konstant zu halten und die Projektlaufzeit zudem gleichzeitig für die OPTINOFA-Entwicklung sowie erste Datenerhebungen effizient zu nutzen.

## Studiendesign und Methoden

### Intelligenter Assistenzdienst zur strukturierten Ersteinschätzung – OPTINOFA

Im Projekt OPTINOFA wurde ein neues, 5‑stufiges Triageinstrument zur strukturierten Ersteinschätzung von Notfällen für die 20 häufigsten notfallmedizinischen Leitsymptome [11] auf Basis der aktuellen Leitlinien und Standard Operating Procedures [2] entwickelt. Hierbei erfolgte für jede Triagestufe neben der Bewertung der Behandlungsdringlichkeit die Zuordnung der erforderlichen Versorgungsstufe: rot = vital bedrohlicher Notfall (sofort/Notaufnahme stationär) – orange = potenziell vital bedrohlicher Notfall (sehr dringlich/Notaufnahme stationär) – gelb = schwerwiegender Notfall (dringlich/Notaufnahme stationär) – grün = nicht schwerwiegender Notfall (zeitverzögert/Notaufnahme ambulant) – blau = kein Notfall (nicht dringlich/niedergelassener Arzt od. kassenärztlicher Bereitschaftsdienst [BD]). Die Notfallalgorithmen wurden digitalisiert, webbasiert im interoperablen Format zur Verfügung gestellt und in 3 Modellkliniken validiert [15].

### Clusterrandomisierte kontrollierte multizentrische Studie im Stepped-wedge-Design

Zur Erprobung von OPTINOFA wurde eine clusterrandomisierte, kontrollierte Studie im Stepped-Wedge Design mit externer Kontrollbedingung unter Beteiligung von 11 Modellkliniken durchgeführt (Onlinesupplement 1): Universitätskliniken Göttingen (UMG), Berlin, Jena, Freiburg, Bonn; Kliniken München-Bogenhausen, Fürth, Wolfsburg, Braunschweig, Wolfenbüttel, Göttingen-Weende.

Das clusterrandomisierte Stepped-wedge-Design wurde für 8 der 11 Modellkliniken (*Cluster-I-Modellklinken*) umgesetzt. Dabei wechselten jeweils 4 Modellkliniken in 2 Schritten von einem 11- bis 12-monatigen Kontroll- zu einem ebenso langen Interventionszeitraum (Datenerhebungszeitraum vom 01.07.2019 bis zum 31.05.2021; 23 Monate). Der Zeitpunkt des Übergangs in den Interventionszeitraum (Schritt 1 oder 2) wurde mittels Blockrandomisierung randomisiert (4-4).

Drei Modellkliniken nutzten OPTINOFA aufgrund struktureller Hürden nicht zur Patientensteuerung (*Cluster-II-Modellkliniken*, nichtrandomisiert). Von diesen wurde nur eine Modellklinik zur Kontrolle von (pandemiebedingten) Zeiteffekten genutzt, da die anderen beiden Modellkliniken nicht oder nur zeitweise in die vertragsärztliche Versorgung verweisen konnten und somit keine adäquate Kontrolle von allgemeinen Zeitraumeffekten ermöglichten.

Als weitere Vergleichskollektive für zeitliche Veränderungen ohne Einsatz von OPTINOFA dienten aggregierte Daten des AKTIN-Notaufnahmeregisters [4] sowie die Routinedaten von AOK-Versicherten des Wissenschaftlichen Instituts der AOK (WIdO).

Die Einschlusskriterien umfassten: Alter ≥ 18 Jahre, Notfallpatient:innen mit einem der 20 häufigsten Leitsymptome. Patient:innen unter 18 Jahren sowie ohne eines der definierten Leitsymptome wurden ausgeschlossen. Die Rekrutierung erfolgte durch das Studienpersonal (Triage Nurses, Prüfärzt:innen) der Modellkliniken und wurde nur während der regulären Öffnungszeiten der Bereitschaftsdienstpraxen (in der Regel Mo, Di, Do 19.00 bis 21:00 Uhr, Mi, Fr 15.00 bis 21.00 Uhr; Sa, So, Feiertage 8.00 bis 21.00 Uhr) durchgeführt.

Der originär im Studienprotokoll definierte primäre Endpunkt umfasste den Anstieg des Anteils der stationär aufgenommenen Notfälle sowie die konsekutive Reduktion des Anteils ambulanter Notfallbehandlungen in der Notaufnahme im Vergleich zwischen Kontroll- und Interventionszeitraum. Die Untersuchung dieses Endpunkts setzt jedoch eine Stabilität des Notfallpatientenkollektivs voraus, die im Studienzeitraum bedingt durch die Coronapandemie nicht gegeben war. Daher wurde die Operationalisierung des primären Endpunkts angepasst und der Anteil der Zuweisungen in den ambulanten Sektor analysiert, der mit Einsatz von OPTINOFA signifikant steigen sollte. Die sekundären Endpunkte umfassten das Outcome bei Entlassung/Verlegung, Prozessindikatoren (Wartezeit, Verweildauer, diagnostische Übereinstimmung und Effizienz) sowie die Kosten der Notfallbehandlung (Tab. 1).

**Tab. 1:** Operationalisierung der primären und sekundären Endpunkte im Projekt OPTINOFA

Endpunkte
*Primärer Endpunkt: Zuweisung zur Versorgungsstufe*
*Originäre Operationalisierung des primären Endpunkts:*^a^ Der Anteil der stationär aufgenommenen Notaufnahmevorstellungen an allen innerhalb der Notaufnahme behandelten Fälle steigt vom Kontrollzeitraum zum Interventionszeitraum; der Anteil ambulanter Notfallbehandlungen in der Notaufnahme sinkt konsekutiv
*Aufgrund der Auswirkungen der COVID-19-Pandemie angepasste Operationalisierung des primären Endpunkts:* Der Anteil der Notaufnahmevorstellungen, die in die vertragsärztliche Versorgung verwiesen werden, steigt vom Kontrollzeitraum zum Interventionszeitraum
*Sekundäre Endpunkte*
*Outcome der Notfallbehandlung bei ambulantem Verbleib*: Anteil der Patientinnen und Patienten, die aufgrund des Erstkontakts in der Notaufnahme nicht unmittelbar stationär aufgenommen werden (also ambulant behandelt oder in die vertragsärztliche Versorgung geleitet werden), aber nach Entlassung aus der Notaufnahme innerhalb von maximal 3 Tagen nach Erstkontakt mit der Notaufnahme stationär aufgenommen werden, bleibt mit dem Einsatz des OPTINOFA-Assistenzdiensts im Vergleich zum Kontrollzeitraum stabil
*Reduktion der Wartezeiten* von Ankunft in der Notaufnahme (Einlesen der elektronischen Gesundheitskarte) bis zum ersten Behandlungskontakt vom Kontroll- zum Interventionszeitraum
*Reduktion der Verweildauer* in der Notaufnahme zwischen Kontroll- und Interventionszeitraum für Patientinnen und Patienten, die stationär aufgenommen werden, definiert als Zeitintervall von der Registrierung in der Notaufnahme (Einlesen der elektronischen Gesundheitskarte) bis zur Verlegung
*Verbesserung der diagnostischen Übereinstimmung und Effizienz *zwischen Kontroll- und Interventionszeitraum für Patientinnen und Patienten, die stationär aufgenommen werden, (diagnostische Übereinstimmung = Übereinstimmung von Hauptdiagnose (ICD-Dreisteller) der Notaufnahme und Entlassdiagnose der stationären Behandlung; diagnostische Effizienz = diagnostische Übereinstimmung/Verweildauer)
*Reduktion der mittleren Kosten der Notfallbehandlung *vom Kontroll- zum Interventionszeitraum bei allen Patientinnen und Patienten mit Erstkontakt in der Notaufnahme (Summe der Kosten über alle Personen, die in der Notaufnahme bzw. im vertragsärztlichen Bereitschaftsdienst versorgt werden, einschließlich der über 28 Tage eventuell anfallenden Kosten einer ambulanten bzw. stationären Versorgung mit Aufnahmedatum innerhalb des Zeitintervalls Erstkontakt plus 3 Tage)

^a^Originäre primäre Endpunkte: 1. Der Anteil der ambulanten Notfallbehandlungen in den Notaufnahmen wird vom Kontrollzeitraum zum Interventionszeitraum um 30 % reduziert (d. h., der Anteil sinkt von aktuell durchschnittlich 60 % auf ca. 51 %), da durch die verbesserte Steuerung der Anteil der Fälle mit leichteren Notfällen, die auch vertragsärztlich ambulant behandelt werden können, in der Notaufnahme sinkt. 2. Der Anteil der Fälle in den Notaufnahmen, die stationär aufgenommen werden, steigt in der Folge vom Kontrollzeitraum zum Interventionszeitraums von heute ca. 40 % auf durchschnittlich ca. 49 %

Nach Erhalt des positiven Ethikvotums (UMG, Antragsnr. 22/12/18) wurde die elektronische „case report form“ (eCRF) mittels der Studiendatenbanksoftwares secuTrial© (InterActive Systems GmbH, Quintessenz Verlags GmbH, Berlin, Deutschland) bzw. OpenClinica© (Needham, MA, USA) in den Modellkliniken implementiert. Routinedaten der beteiligten Krankenkassen (AOK Niedersachsen, DAK, TK, IKK classic, Audi BKK) zu den Kosten der Notfallbehandlung innerhalb von 28 Tagen nach Einschluss in OPTINOFA sowie zum Verlauf wurden fallbezogen erhoben und mittels „data linkage“ verknüpft.

### Evaluation und Statistik

Als Datengrundlage für die Deskription und Inferenzstatistik wurden die eCRF-Daten von 8 Cluster-I- und einer Cluster-II-Modellklinik sowie die Routinedaten der Krankenkassen verwendet.

Die Datenanalyse erfolgte separat für Cluster-I- und Cluster-II-Modellkliniken mittels der Software R. Für alle Cluster-I-Modellkliniken wurde die Veränderung über die Zeit (Interventionseffekt = Unterschied zwischen Kontroll- und Interventionszeitraum) in „mixed models“ modelliert, sodass die Heterogenität der Modellkliniken im Mittelwert des Endpunkts („random intercept“) und im Interventionseffekt („random slope“) berücksichtigt wurde. Je nach Skalierung des Endpunkts wurden lineare oder logistische „mixed models“ genutzt. Da die Anwendung des OPTINOFA-Assistenzdiensts nicht in allen 8 Cluster-I-Modellkliniken stringent und damit protokollgerecht durchgeführt wurde, erfolgte jeweils eine separate Analyse der Daten in 2 sog. Per-protocol-Modellkliniken.

Der um allgemeine Zeiteffekte bereinigte Interventionseffekt wurde schließlich jeweils durch den Unterschied in den prädizierten Werten im Kontroll- und Interventionszeitraum zwischen den Cluster-I-Modellklinken (bzw. Per-protocol-Cluster-I-Kliniken) und der Cluster-II-Modellklinik berechnet und grafisch veranschaulicht. Alle Analysen wurden für die Fallmerkmale Alter, Geschlecht, Behandlungsdauer des Leitsymptoms, stationäre Aufnahmewahrscheinlichkeit des Leitsymptoms, Triagestufe und geschlossene BD-Praxen zum Vorstellungszeitpunkt adjustiert (Signifikanzniveau = 5 %).

Zur Kontrolle der zeitlichen Effekte (insbes. Pandemieeffekte) wurden zudem die Daten des AKTIN-Notaufnahmeregisters [4] aus *n* = 7 Kliniken (Fallzahl *n* = 173.350) sowie die Daten des WiDO-Vergleichskollektivs (Fallzahl *n* = 9.406.450) im gleichen Erhebungszeitraum (Onlinesuppl. 1, Abb. 2) als Kontrolldaten analysiert, um zur Evaluation der tatsächlichen Interventionseffekte die Zeiteffekte (Pandemieeffekte) sowie Effekte durch die Heterogenität der Modellkliniken weitgehend zu kontrollieren.

Die BD-Praxen wurden im Rahmen der Coronapandemie zeitweilig geschlossen. Da die klinische Studie hiervon insbesondere im Interventionszeitraum (01.06.2020 bis 31.05.2021) betroffen war, wurden alle Fälle, die zum Zeitpunkt geschlossener BD-Praxen in den Notaufnahmen in die Studie eingeschlossen wurden, in den Analysen nicht berücksichtigt.

## Ergebnisse

In die OPTINOFA-Studie wurden im Kontrollzeitraum *n* = 46.558 und im Interventionszeitraum *n* = 37.485 Notfälle eingeschlossen (Flowchart, Onlinesuppl. 1).

### Strukturierte Ersteinschätzung mittels des intelligenten Assistenzdienstes OPTINOFA

In den Cluster-I-Modellkliniken erhielten mittels OPTINOFA drei Viertel der Notfälle eine Empfehlung für die Zuweisung zur stationären Versorgung, ein Viertel zur ambulanten Behandlung (Tab. 2).

**Tab. 2:** OPTINOFA-Triagestufen in den Cluster-I-Modellkliniken

OPTINOFA-Empfehlung				95 %-KI	
Dringlichkeit	Versorgungsstufe	*n*	%	LO (in %)	UP (in %)
Rot	NA stationär	1051	3,99	3,76	4,23
Orange		7099	26,95	26,42	27,49
Gelb		11.516	43,72	43,13	44,32
Grün	NA ambulant	4669	17,73	17,27	18,19
Blau	BD-Praxis	1997	7,58	7,27	7,91
Fehlend	–	6	–	–	–

**Abb. 1:**
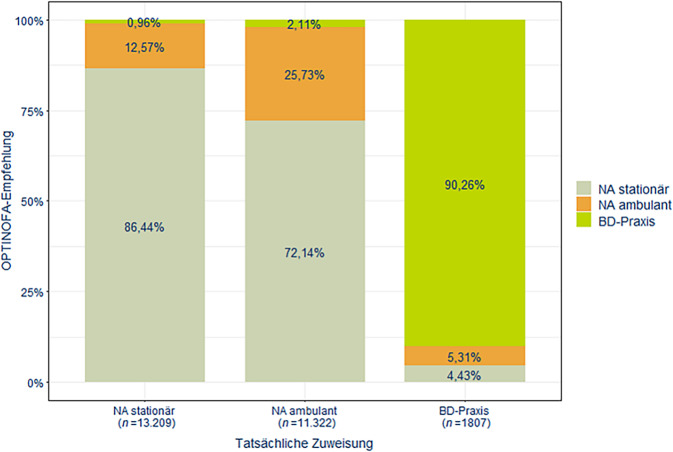
Übereinstimmung von OPTINOFA Empfehlung und tatsächlicher Zuweisung zur Versorgungsstufe im Cluster‑I

Eine hohe Übereinstimmung der gemäß OPTINOFA empfohlenen Versorgungsstufe und der tatsächlichen Versorgungsstufe zeigte sich für stationäre Notfälle (86,44 %) sowie für die an eine BD-Praxis verwiesenen Fälle (90,26 %; Abb. 1). Diese Quote lag für ambulant in der Notaufnahme behandelte Fälle deutlich niedriger (25,73 %). Es zeigte sich eine moderate bis starke Korrelation zwischen der OPTINOFA-Empfehlung und der Disposition (tatsächliches Versorgungsziel; ρ = 0,40; *p* < 0,001; Onlinesuppl. 2: Abb. 1).

**Abb. 2:**
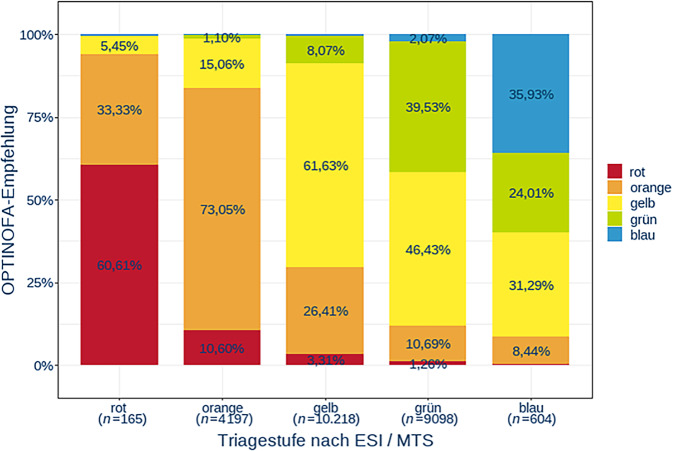
Übereinstimmung von ESI- bzw. MTS-Triagestufe und OPTINOFA-Stufe im Cluster I

Der Vergleich von OPTINOFA-Stufe und ESI- bzw. MTS-Triagestufe ergab in den Stufen rot, orange und gelb eine höhere Übereinstimmung (ca. 60 % rot, gelb und 73 % Orange) als bei grün (39,53 %) und blau (35,93 %; Abb. 2). Dabei zeigte sich für OPTINOFA ein Trend zur Zuweisung einer Stufe mit höherer Behandlungsdringlichkeit. Signifikante Unterschiede zwischen ESI und MTS ergaben sich dabei nicht.

### Primärer Endpunkt

Für den aufgrund der Pandemiebedingungen angepassten primären Endpunkt ergab sich in den Per-protocol-Kliniken ein deutlicher Anstieg der Zuweisung in die BD-Praxen: So erhielten in diesen Modellkliniken im Kontrollzeitraum 12,26 % der Fälle und im Interventionszeitraum in 30,4 % der Fälle eine Zuweisung in die BD-Praxen (RR = 2,48). Bei isolierter Betrachtung der Fälle mit den Dringlichkeitsstufen grün bzw. blau ergab sich in den Per-protocol-Modellkliniken sogar ein Anstieg der Verweisrate von 48,18 % im Kontrollzeitraum auf 78,17 % im Interventionszeitraum (OR = 3,85) und in allen Cluster-I-Modellkliniken ein Anstieg von 8,58 auf 15,67 % (OR = 1,97; Onlinesuppl. 2: Tab. 1). In der Cluster-II-Modellklinik kam es demgegenüber zu einem geringeren Anstieg von 39,51 auf 47,67 %.

**Abb. 3:**
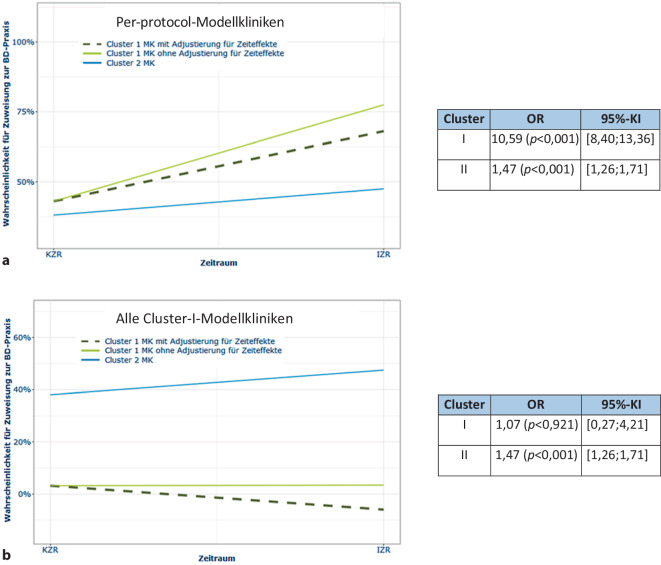
**a** Signifikanter Anstieg der Zuweisung in die BD-Praxen im Interventionszeitraum im Vergleich zum Kontrollzeitraum in den Per-protocol-Kliniken. **b** Zuweisung in die BD-Praxen im Interventionszeitraum im Vergleich zum Kontrollzeitraum in allen Cluster-I-Kliniken

In der Inferenzstatistik fand sich dementsprechend für die Per-protocol-Kliniken im Interventionszeitraum ein signifikanter Anstieg der Zuweisungen in die BD-Praxen im Vergleich zum Kontrollzeitraum und zwar sowohl mit als auch ohne Adjustierung für die Zeiteffekte (Abb. 3a). Der Anstieg der Verweisrate war für alle Cluster-I-Modellkliniken (Abb. 3b) nicht signifikant.

Für den originären primären Endpunkt zeigten sich deskriptiv und in der Inferenzstatistik kaum Veränderungen zwischen Kontroll- und Interventionszeitraum im Anteil der stationären oder ambulanten Zuweisungen in der Notaufnahme, weder in allen Cluster-I-Modellkliniken noch in der Cluster-II-Modellklinik (Onlinesuppl. 2: Tab. 2, Abb. 2).

### Stabiles Outcome der Notfallbehandlung bei ambulantem Verbleib

In der Deskription kam es in den Per-protocol-Kliniken und den Cluster-I-Modellkliniken sowie der Cluster-II-Modellklinik zu einem leichten Anstieg des Anteils ambulanter Notfallpatient:innen, die innerhalb von 3 Tagen nach der Notfallbehandlung doch vollstationär im Krankenhaus aufgenommen werden mussten (Onlinesuppl. 2: Tab. 3).

**Abb. 4:**
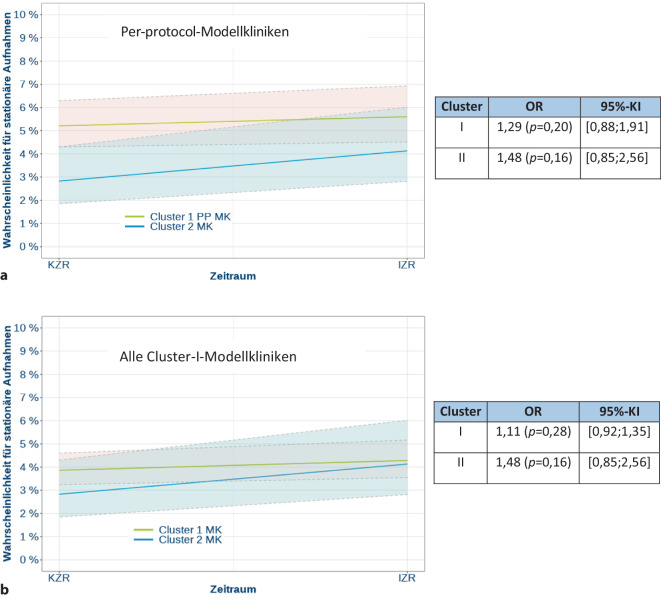
**a** Inferenzstatistik des Outcome der Notfallbehandlung in Bezug auf die Wahrscheinlichkeit für eine stationäre Aufnahme innerhalb von 3 Tagen nach ambulanter Notfallbehandlung in den Per-protocol-Modellkliniken. **b** Inferenzstatistik des Outcome der Notfallbehandlung in Bezug auf die Wahrscheinlichkeit für eine stationäre Aufnahme innerhalb von 3 Tagen nach ambulanter Notfallbehandlung in allen Cluster-I-Modellkliniken

Die Inferenzstatistik zeigte weder für die Per-protocol-Kliniken (Abb. 4a) noch für die Cluster-I-Modellkliniken (Abb. 4b) eine signifikante Veränderung in der Wahrscheinlichkeit für eine stationäre Aufnahme vom Kontroll- zum Interventionszeitraum. Bei der oberen Grenze des Konfidenzintervalls zeigte sich hier jeweils ein Unterschied von maximal kleiner Effektstärke.

### Reduktion der Wartezeiten in der Notaufnahme

Für die Wartezeit bis zum ersten Arztkontakt ergab sich deskriptiv in den Per-protocol-Modellkliniken eine deutliche Reduktion um fast 20 min, in allen Cluster-I-Modellkliniken von 11 min. Demgegenüber reduzierte sich die Wartezeit in der Cluster-II-Modellklinik nur um knapp 5 min (Onlinesuppl. 2: Tab. 4).

**Abb. 5:**
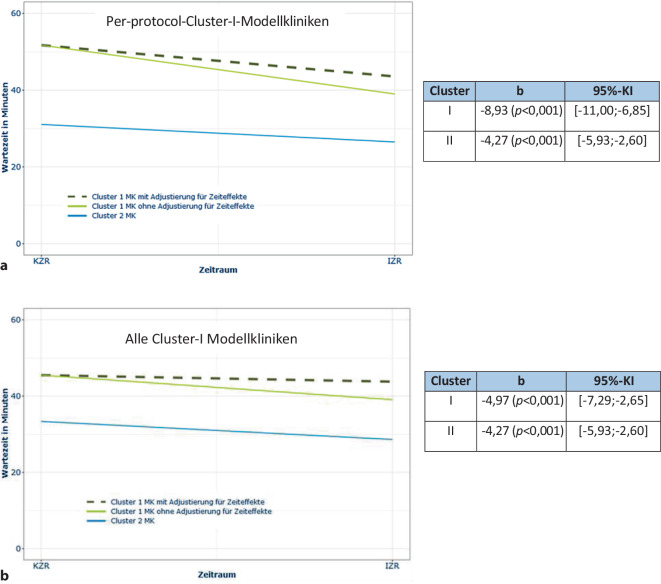
**a** Inferenzstatistik der Wartezeiten in den Per-protocol-Modellkliniken. **b** Inferenzstatistik der Wartezeiten in allen Cluster-I-Modellkliniken

In der Inferenzstatistik zeigte sich diese Reduktion der Wartezeiten in der Notaufnahme im Interventionszeitraum nach Adjustierung der Zeiteffekte in den Per-protocol-Modellkliniken (Abb. 5a) und zwar noch stärker als in den Cluster-I-Modellkliniken (Abb. 5b).

### Reduktion der Verweildauer in der Notaufnahme

Die Verweildauer von stationären Fällen in der Notaufnahme zeigte deskriptiv ein inkonsistentes Ergebnis: Während in allen Cluster-I-Modellkliniken die Verweildauer vom Kontrollzeitraum zum Interventionszeitraum um ca. 10 min abnahm, war in den Per-protocol-Modellkliniken (+41,69 min), der Cluster-II-Modellklinik (+35,66 min) und den AKTIN-Kliniken (+6,51 min) ein leichter Anstieg der Verweildauer zu beobachten (Onlinesuppl. 2: Tab. 5).

**Abb. 6:**
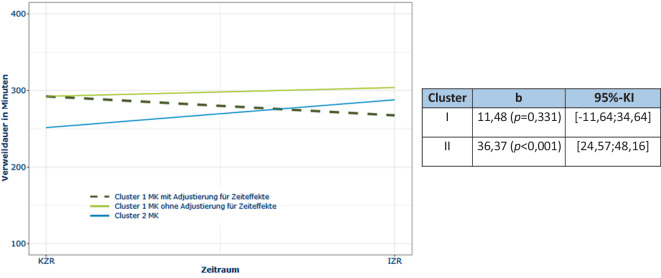
Inferenzstatistik der Verweildauer in der Notaufnahme adjustiert um Zeiteffekte für alle Cluster-I-Modellkliniken

In der Inferenzstatistik zeigte sich für alle Cluster-I-Modellkliniken nach Adjustierung für allgemeine Zeiteffekte demgegenüber eine Reduktion der Verweildauer (Abb. 6).

### Reduktion der mittleren Kosten der Notfallbehandlung

Im Cluster I und der Cluster-II Modellklinik ergab sich ein geringer Anstieg der Kosten. Bei ausschließlicher Betrachtung der Kosten nach initial ambulanter Zuweisung fand sich deskriptiv für alle Cluster-I-Modellkliniken und die Per-protocol-Modellkliniken eine Kostenzunahme von ca. 60 € bzw. 110 €, jedoch in der Cluster-II-Modellklinik eine deutlichere Kostensteigerung von knapp 350 €. In der Deskription ergab sich demgegenüber für die Per-protocol-Modellkliniken eine Abnahme der Kosten vom Kontrollzeitraum zum Interventionszeitraum um −273 € pro Fall innerhalb von 28 Tagen nach Studieneinschluss. Dies beruhte auf einer Reduktion der stationären Leistungsausgaben (Onlinesuppl. 2: Tab. 6).

**Abb. 7:**
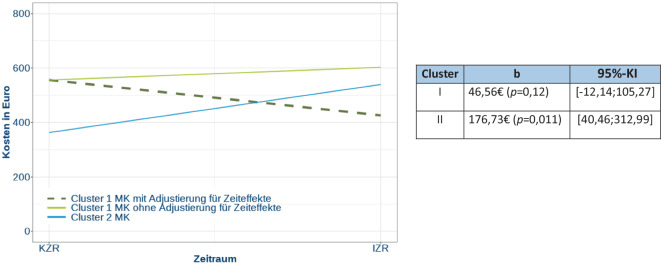
Inferenzstatistik der mittleren Kosten der Notfallbehandlung in allen Cluster-I-Modellkliniken bei ambulanten Fällen nach Adjustierung der Zeiteffekte

Die Inferenzstatistik für die initial ambulant zugewiesenen Fälle zeigte nach Adjustierung der Zeiteffekte für alle Cluster-I-Modellkliniken eine relative Kostenreduktion (Abb. 7). Für die beiden Per-protocol-Modellkliniken ergab sich kein einheitlicher Trend, aber insbesondere kein Hinweis für eine Kostensteigerung.

## Diskussion

Mit OPTINOFA steht in Deutschland erstmalig ein digitaler Assistenzdienst zur strukturierten Ersteinschätzung von Behandlungsdringlichkeit und erforderlicher Versorgungsstufe in der Notfallversorgung zur Verfügung. Mit OPTINOFA wird eine transsektorale Steuerung der Patientenströme in der Notaufnahme erzielt und damit ein zentrales Problem im Status quo der Notfallversorgung [20] adressiert.

Zur Entwicklung wurden die Inhalte der etablierten Triagesysteme zugrunde gelegt und evidenzbasiertes Expertenwissen für einen neuen OPTINOFA-Algorithmus integriert, um eine zeitgleiche Zuordnung von Behandlungsdringlichkeit und Versorgungsstufe in der Ersteinschätzung zu ermöglichen.

Die Erprobung von OPTINOFA erfolgte in einer klinischen Studie in 8 Modellkliniken. Hierbei handelt es sich um die erste bundesweite Studie, die unter Einhaltung der wissenschaftlichen Gütekriterien der Randomisierung und Kontrolle sowie im multizentrischen Design mit > 45.000 Fällen im Kontrollzeitraum und > 35.000 im Interventionszeitraum durchgeführt wurde.

Für den *primären Endpunkt* konnte bei stringenter, protokollgerechter Anwendung der strukturierten Ersteinschätzung mittels OPTINOFA ein signifikanter Anstieg der Weiterleitung von Notfallpatient:innen in den ambulanten, vertragsärztlichen Bereich und damit eine transsektorale Steuerung der Notfallpatient:innen in der Notaufnahme erzielt werden.

In der Anwendung von OPTINOFA ergab sich dabei eine gute Vergleichbarkeit mit den etablierten Triagesystemen in Bezug auf die Disposition. Die Ersteinschätzung mittels OPTINOFA tendierte zwar zu einer höheren Stufe der Behandlungsdringlichkeit, die jedoch unter Berücksichtigung des obersten Primats der Patientensicherheit in der Notfallversorgung nicht negativ zu bewerten ist. Unter Berücksichtigung der Öffnungszeiten und Strukturvoraussetzungen ist jedoch der Anteil von Patient:innen, der in die vertragsärztliche Behandlung geleitet werden kann, trotz Anstieg mit ca. 7 % aktuell relativ gering.

Für den originären primären Endpunkt der Studie zeigte sich der erwartete signifikante Anstieg der stationären Zuweisungen in den Cluster-I-Modellkliniken bzw. die konsekutive Reduktion der ambulanten Notfallbehandlungen in der Notaufnahme nicht. Dieser originäre Endpunkt aber setzt eine Stabilität des Notfallpatientenkollektivs voraus, die aufgrund der Einflüsse der Coronapandemie im Studienzeitraum nicht gegeben war. Daher wurde die Operationalisierung des primären Endpunkts angepasst und zur Bewertung des primären Endpunkts der Anteil der Notfälle mit Verweis in die ambulante, vertragsärztliche Versorgung analysiert, der durch den Einsatz von OPTINOFA signifikant steigen sollte. Dieser angepasste primäre Endpunkt wurde in der Studie erreicht.

In Hinsicht der sekundären Endpunkte fand sich bezüglich des *Outcome* kein Anhalt für einen Anstieg der stationären Aufnahmerate innerhalb von 3 Tagen nach initial ambulanter Notfallbehandlung. Daher ist mit Anwendung von OPTINOFA die Patientensicherheit trotz Zuweisung in den ambulanten Sektor gewährleistet.

In Bezug auf die *Wartezeiten* fand sich bei den Cluster-I-Modellkliniken eine signifikante Reduktion. Hieraus lässt sich ableiten, dass der Einsatz von OPTINOFA auch zu einer Optimierung der Prozesszeiten unmittelbar nach Durchführung der strukturierten Ersteinschätzung führt.

Demgegenüber ergab sich in der Analyse der *Verweildauer* ein inkonsistentes Studienergebnis. Für diesen sekundären Endpunkt ist jedoch zu beachten, dass bei stationären Aufnahmen der sog. Exit-Block [13] einen relevanten Einflussfaktor darstellt [17], insbes. in der Coronapandemie. Da ein Anstieg der Verweildauer auch in den AKTIN- und Cluster-II-Kliniken zu beobachten war, ist der Parameter „Verweildauer“ daher nicht gut geeignet, den potenziellen Interventionseffekt auf die Performance innerhalb der Notaufnahme zu beurteilen. Ein besserer Indikator wäre hierfür die originäre Dauer der Notfallbehandlung operationalisiert über das Zeitintervall zwischen den Zeitstempeln „erster Arzt/Pflegekontakt“ und „Ende der Notfallbehandlung“.

In Bezug auf die *Kosten* der Notfallbehandlung ergab sich bei einer isolierten Betrachtung der ambulanten Fälle für die Cluster-I-Modellkliniken eine relative Kostenreduktion im Vergleich zu der Cluster-II-Modellkliniken, während es für die Per-protocol-Modellkliniken zumindest nicht zu einer Kostensteigerung kam. Hieraus ergibt sich der Hinweis auf Einsparungen von Leistungsausgaben bei initial ambulanter Notfallbehandlung im Vergleich zu den allgemeinen zeitlichen Trends durch den Einsatz von OPTINOFA.

### Limitationen

1. *Coronapandemie*: Die Studie wurde am Ende des Kontrollzeitraums und im gesamten Interventionszeitraum unter den Bedingungen der Coronapandemie durchgeführt. Daher müssen die zahlreichen Pandemieeffekte, u. a. Fallzahlen, Leitdiagnosen- und Triagestufenspektrum der Patientenkollektive, Strukturen in den Modellkliniken (Schließung BD-Praxen; [3, 22, 23]), bei der Bewertung der Ergebnisse berücksichtigt werden. Dies ist u. a. durch die Adjustierung allgemeiner Zeiteffekte und der Schließungszeiten der BD-Praxen sowie der Anpassung der Fallzahlen erfolgt. Zudem wurde aus diesem Grund die Operationalisierung des primären Endpunkts angepasst, sodass pandemiebedingte Veränderungen in der Zusammensetzung des Patientenkollektivs einen geringeren Einfluss auf die Ergebnisse haben sollten.
2. *Heterogenität der Modellkliniken*: Die Strukturdaten der Modellkliniken zeigten bereits initial eine hohe Heterogenität bezüglich der Anbindung des ambulanten Sektors, sodass eine Clusterung erfolgte. Da im Cluster II jedoch nur eine Modellklinik im gesamten Beobachtungszeitraum ein etabliertes Verweissystem nutzte, konnten für die Adjustierung der Zeiteffekte ausschließlich Daten dieser Modellklinik verwendet werden. Modellklinikspezifische und regionale Einflüsse auf die Analysen können daher nicht ausgeschlossen werden. Darüber hinaus schwankte das Verweispotenzial von ambulanten Notfällen in die BD-Praxis zwischen den Cluster-I-Modellkliniken sehr stark. Auch zeigte sich in den Monitoringvisiten, dass OPTINOFA nicht in allen Modellkliniken stringent und protokollgerecht durchgeführt wurde; daher ist die Evaluation in den Per-protocol-Kliniken hoch relevant.

Zusammenfassend ist es im OPTINOFA-Projekt gelungen, einen validen Assistenzdienst zur strukturierten Ersteinschätzung von Behandlungsdringlichkeit und Versorgungsstufe in der Notaufnahme zu entwickeln und technisch als digitales Triageinstrument auf mobilen Endgeräten im interoperablen Format zur Verfügung zu stellen. Die Studienergebnisse belegen ein großes Potenzial für den OPTINOFA-Assistenzdienst hinsichtlich der transsektoralen Steuerung der Patientenströme in der Notfallversorgung. Zur weiteren Entwicklung soll OPTINOFA für alle notfallmedizinischen Leitsymptome erweitert, in enger Kooperation mit den Fachgesellschaften und dem vertragsärztlichen Sektor iterativ optimiert und schließlich für den Einsatz in den zukünftigen integrierten Notfallzentren evaluiert werden. Für die Zertifizierung als Medizinprodukt ist eine weitere klinische Studie in Vorbereitung.

## Fazit für die Praxis

- Mit OPTINOFA steht ein valider Assistenzdienst als digitales Triageinstrument zur strukturierten Ersteinschätzung von Behandlungsdringlichkeit und Versorgungsstufe in der Notaufnahme zur Verfügung.
- Die Ergebnisse der multizentrischen Studie belegen ein großes Potenzial für den OPTINOFA-Assistenzdienst hinsichtlich der transsektoralen Steuerung der Patientenströme in der Notfallversorgung.
- Zur weiteren Entwicklung soll OPTINOFA für alle notfallmedizinischen Leitsymptome erweitert und für den Einsatz in den zukünftigen integrierten Notfallzentren als Medizinprodukt zertifiziert werden.

## Supplementary Information

Supplement 1 Studiendesign OPTINOFA

Supplement 2 Weitere Ergebnisse

## Data Availability

Weitere Studienergebnisse können beim korrespondierenden Autor angefordert werden.
