# Supplementary material for: Structured triage in the emergency department via intelligent assistant service OPTINOFA: Results of a multicenter, cluster-randomized and controlled interventional study in Germany
Source: Med Klin Intensivmed Notfmed. 2024 Dec 16;120(7):585–95. [Article in German] doi: 10.1007/s00063-024-01229-6 (PMC12504124; doi:10.1007/s00063-024-01229-6)
Supplement: Supplementary file 1 — Supplement 1 Studiendesign OPTINOFA [file 63_2024_1229_MOESM1_ESM.docx]

**Supplement 1** Studiendesign OPTINOFA

*Multizentrische, Cluster-randomisierte und kontrollierte Interventionsstudie im Stepped-Wedge Design*

Zur Erprobung des intelligenten Assistenzdienstes OPTINOFA in der strukturierten Ersteinschätzung von Notfällen wurde im Rahmen des Projekts eine Cluster-randomisierte, kontrollierte klinische Studie im Stepped-Wedge-Design mit externer Kontrollbedingung unter Beteiligung der Zentralen Notaufnahmen von elf **Modellkliniken** durchgeführt: Universitätsmedizin Göttingen (UMG), Charité-Universitäts-medizin Berlin, Universitätsklinik Jena, Universitätsklinik Freiburg, Universitätsklinik Bonn, München-Klinik Bogenhausen, Klinikum Fürth, Klinikum Wolfsburg, Städtisches Klinikum Braunschweig, Städtisches Klinikum Wolfenbüttel, Evangelisches Krankenhaus Göttingen-Weende. Hierbei wurden Zentrale Notaufnahmen in Krankenhäusern der Maximalversorgung mit integrierter oder enger Anbindung einer kassenärztlichen Bereitschaftsdienstpraxis in möglichst verschiedenen Bundesländern ausgewählt^[[1]](#footnote-1)^. Nach Erhebung der Strukturdaten in den beteiligten Modellkliniken ergab sich, dass drei Modellkliniken (Bonn, Jena, Berlin) die OPTINOFA Empfehlungen nicht umsetzen konnten. Daher erfolgte eine Gruppierung der Modellkliniken in acht Cluster-I Modellkliniken, die OPTINOFA einsetzten und den Verweis in die vertragsärztliche Praxis aktiv durchführten, und drei Cluster-II Modellkliniken, die OPTINOFA zwar einsetzten, die Empfehlung aber nicht umsetzten.

Nach Erstellung des **Studienprotokolls** und des Datenschutzkonzepts wurde ein positives Ethikvotum der federführenden Ethikkommission in der UMG eingeholt (Antragsnr. 22/12/18) und die elektronische Case Report Form (eCRF) mittels Studiendatenbank-Software secuTrial^©^ bzw. Openclinica^©^ in den Modellkliniken implementiert. Im eCRF wurden in den beteiligten Notaufnahmen folgende Parameter erfasst: Strukturdaten der Modellkliniken (Notfallstufe, Fachabteilungen, Triage-System, KV-Bereitschaftsdienstpraxis inkl. Öffnungszeiten); Datenerhebung pro Fall (Alter; Geschlecht; Leitsymptom; Zeitstempel: Aufnahme, Triage, Erstkontakt, Entlassung/Verlegung; Triagestufe; OPTINOFA Stufe; Zuweisung zur Versorgungsstufe; Abweichungsgründe; Verlegungsziel). Darüber hinaus wurden seitens der beteiligten Krankenkassen (AOK Niedersachsen, DAK, TK, IKK classic, Audi BKK) Daten zu den Kosten der Notfallbehandlung innerhalb von 28d nach Einschluss in OPTINOFA sowie zu stationären Aufnahmen innerhalb von 3d nach Notaufnahmevorstellung erhoben und mittels Data Linkage mit den Datensätzen der Studiendatenbank verknüpft.

Die **Einschlusskriterien** in die klinische Studie umfassten: Alter ≥ 18 Jahre, Notfallpatient:in mit einem der zwanzig häufigsten Leitsymptome; Patient:innen unter 18 Jahre sowie ohne eines der definierten Leitsymptome wurden ausgeschlossen. Die Rekrutierung erfolgte durch das Studienpersonal (Triage Nurses, Prüfärzt:innen) der Modellkliniken und wurde nur während der regulären Öffnungszeiten der Bereitschaftsdienstpraxen durchgeführt.

Der **primäre Endpunkt** der Studie umfasste den signifikanten Anstieg der Weiterleitung in den ambulanten Sektor. Der Endpunkt beschreibt gemäß Studienprotokoll den Anstieg im Anteil der stationär aufgenommenen Notaufnahmevorstellungen an allen innerhalb der Notaufnahme behandelten Fälle vom Kontrollzeitraum zum Interventionszeitraum. Die Operationalisierung des primären Endpunkts musste aufgrund der Einflüsse der Corona Pandemie auf die Zusammensetzung des Notfallpatientenkollektivs und weiterer pandemiebedingter Auswirkungen auf stationäre und ambulante Behandlungen angepasst werden. Daher wurde zur Überprüfung des primären Endpunkts der Anteil der in die ambulante, vertragsärztliche Versorgung verwiesenen Notfälle analysiert, der durch den Einsatz von OPTINOFA steigen sollte.

Die **sekundären Endpunkte** bezogen sich auf das Outcome der Notfallbehandlung bei Entlassung oder Verlegung, die Prozess- und Qualitätsindikatoren (Wartezeit, Verweildauer, diagnostische Übereinstimmung, diagnostische Effizienz) sowie die mittleren Kosten der Notfallbehandlung.

Die Operationalisierung der Endpunkte der klinischen Studie ist in **Tab. 1** dargestellt.

*Durchführung der klinischen Studie im Stepped-Wedge-Design*

Die Durchführung der Studie erfolgte cluster-randomisiert im Stepped-Wedge-Design über eine Laufzeit von 23 Monaten: Das Stepped-Wedge Design wurde nur für die acht o.g. Cluster-I Modellkliniken umgesetzt, d. h. das Erreichen der primären und sekundären Endpunkte der Effektevaluation von OPTINOFA wurde mittels eines cluster-randomisierten Stepped-Wedge Designs mit den Modellkliniken des Clusters-I überprüft. Das Stepped-Wedge Design erstreckte sich über 23 Monate und sah vor, dass die Modellkliniken über einen Kontrollzeitraum ohne Einsatz des neuen Triage-Systems OPTINOFA (Ist-Zustand) und einen Interventionszeitraum mit Einsatz des neuen Triage-Systems OPTINOFA beobachtet wurden. Dabei implementierten die Modellkliniken nicht alle gleichzeitig, sondern sukzessive in zwei Schritten die neue Versorgungsform, wobei die Reihenfolge randomisiert wurde: Die Blockrandomisierung wurde von einer unabhängigen, nicht mit der Datenerhebung befassten Stelle (Evaluator IGES Institut) mittels R-Paket blockrand mit gleicher Anzahl an Modellkliniken in den zwei möglichen Schritten durchgeführt. Die Cluster-I Modellkliniken wurden dabei nach dem Kontrollzeitraum zu unterschiedlichen Zeitpunkten in den Interventionszeitraum überführt. Dementsprechend wurden zwei Gruppen (A,B) von Cluster-I Modellkliniken (4-4) randomisiert, die jeweils zeitversetzt um einen Monat in den Interventionszeitraum gestartet sind. Nach einem initialen 11-monatigen Kontrollzeitraum wurde in vier randomisierten Modellkliniken (Schritt 1, Cluster-IA) die neue Versorgungsform eingeführt. Nach einem weiteren Monat (13. Monat) erhielten vier weitere randomisierte Modellklinken (Schritt 2, Cluster-IB) die neue Versorgungsform (Cluster-Randomisierung; 4-4). Der jeweils letzte Monat des Kontrollzeitraums wurde als Implementierungsphase zur Schulung der Anwender durch die Studienkoordination in der Universitätsmedizin Göttingen als Studienzentrale genutzt. Damit lagen aus dem Kontrollzeitraum und dem Interventionszeitraum jeweils Daten aus elf bzw. zwölf Monaten vor. Die Datenerhebung erfolgte im Zeitraum von 01.07.2019 – 31.05.2021.

*Datenerhebung in Vergleichskollektiven*

Zusätzlich zu dieser cluster-randomisierten Studie im Stepped-Wedge-Design liegen Daten von den folgenden drei Vergleichskollektiven vor:

- **Daten der Cluster-II Modellkliniken**: Aufgrund der nachträglichen Einteilung der Modellkliniken in Cluster-I und Cluster-II Kliniken konnten die Daten der Cluster-II Modellkliniken, die OPTINOFA nicht eingesetzt haben, zur Kontrolle für allgemeine Zeiteffekte, d. h. für Veränderungen im Verhalten der Notfallpatient:innen zur Inanspruchnahme der Notaufnahme, herangezogen werden: Diese stellten aufgrund der zu den Cluster-I Modellkliniken parallelen Datenerhebung und Operationalisierungen die beste Datenquelle für diese Vergleiche dar. Die Cluster-II Modellkliniken haben über den gesamten Erhebungszeitraum Kontrolldaten erhoben, wurden jedoch nicht randomisiert. Folgende unterschiedliche Gründe wurden für die Nicht-Umsetzung der OPTINOFA-Empfehlung benannt: Bei einer Modellklinik war keine Bereitschaftsdienst-Praxis in der Nähe, so dass kein Verweis möglich war. Bei zwei Modellkliniken wurde nach internen Vorgaben zum ärztlichen Bereitschaftsdienst verwiesen, wobei eine Modellklinik diese internen Vorgaben bereits vor Beginn des Beobachtungszeitraums umsetzte und eine erst während der Datenerhebung damit begonnen hat (Verweis in Bereitschaftsdienst-Praxis ab Februar 2020). Die Daten der Cluster-II MK, die bereits vor Beginn des Beobachtungszeitraums nach internen Vorgaben in die vertragsärztliche Versorgung verwiesen hat, stellte die beste Vergleichsgrundlage für „normale“ zeitliche Veränderungen dar, da einerseits, wie in Cluster-I in die vertragsärztliche Versorgung verwiesen wurde und sich andererseits im Erhebungszeitraum die Bedingungen dafür nicht veränderten. Die Daten dieser Cluster-II Modellklinik wurden daher in den Datenanalysen für die Abschätzung zeitlicher (ggf. pandemiebedingter) Effekte genutzt, die nicht auf OPTINOFA zurückzuführen waren.
- **AKTIN-Daten**: Aggregierte Daten des nationalen Notaufnahmeregisters AKTIN (n=7 beteiligte Notaufnahmen, die nicht an OPTINOFA teilnahmen) wurden im gleichen Beobachtungszeitraum (01.07.2019 bis 31.05.2021) analysiert zur Abschätzung der Repräsentativität der beobachteten Veränderungen für Endpunkte, die mittels der eCRF-Daten beantwortet werden sollten.
- **WIdO-Daten**: Die retrospektiven, bundesweiten Datensätze der AOK-Versicherten im Wissenschaftlichen Institut der AOK (WIdO) im Zeitraum vom 01.07.2018 bis 03.06.2020 dienten zur Abschätzung der Repräsentativität der beobachteten Veränderungen für die Endpunkte, die mit den Routinedaten der beteiligten Krankenkassen beantwortet werden sollten.

Das Stepped-Wegde-Design der Studie (**Abb.1**) inklusive der zusätzlichen Vergleichskollektive sowie das Flow Chart (**Abb.2**) sind in den folgenden Abbildungen dargestellt.


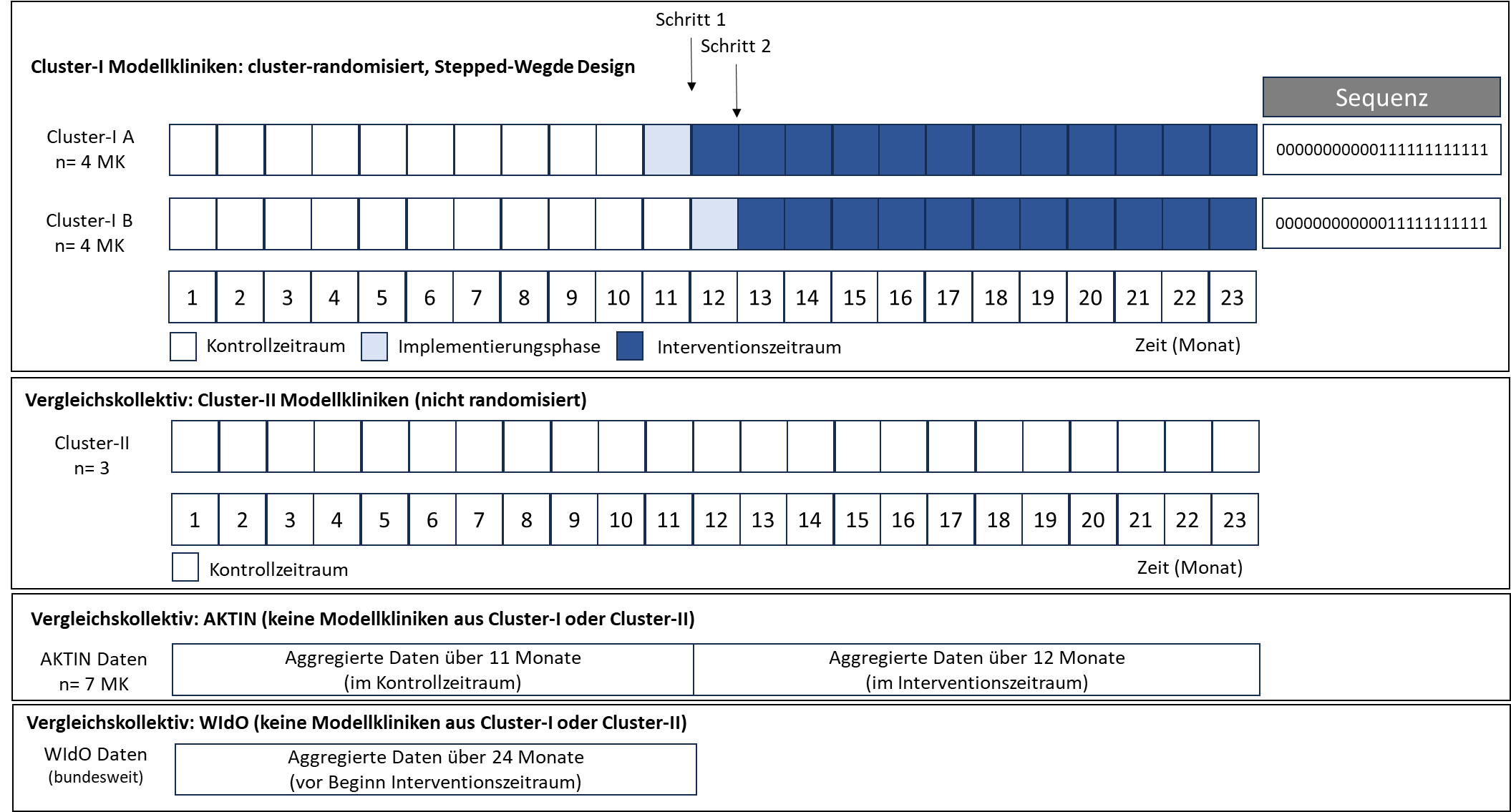


*Abb.1 Design der OPTINOFA Studie – Cluster-randomisiertes Stepped-Wedge-Design in den Cluster-I Modellkliniken und Design in den Vergleichskollektiven*

*(Cluster-II Modellkliniken, AKTIN-Modellkliniken und WIdO)*

**
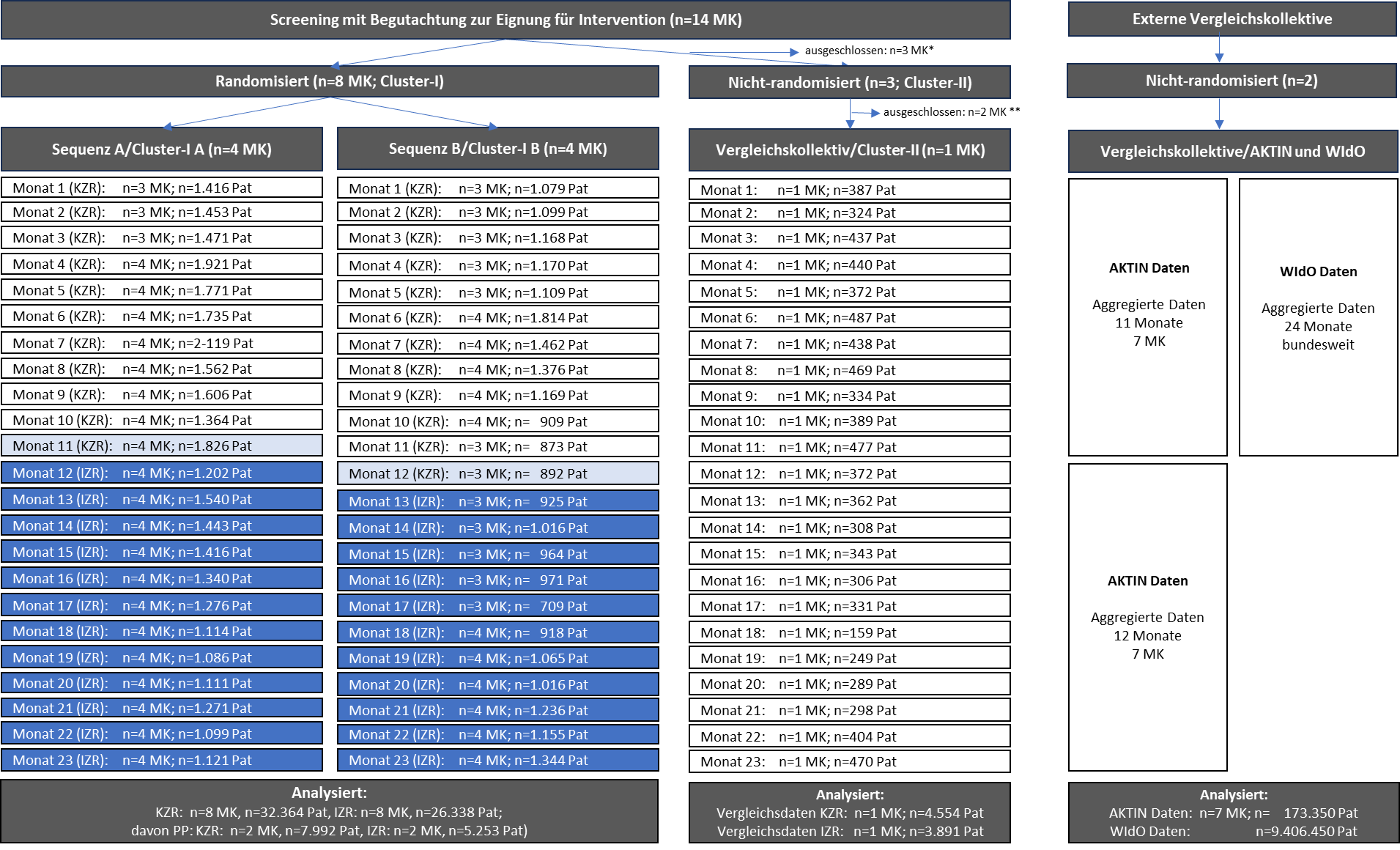
**

KZR Kontrollzeitraum MK Modellkliniken

IZR Interventionszeitraum Pat Notfallpatient:innen

* keine Teilnahme aus organisatorischen Gründen

**kein Verweis in vertragsärztliche Versorgung im gesamten Zeitraum

*Abb. 2 Flow Chart der OPTINOFA Studie*

*Evaluation und Statistik*

Als Datengrundlage für die Analysen wurden die eCRF Daten von acht Cluster-I Kliniken und einer Cluster-II Klinik sowie die Routinedaten der beteiligten Krankenkassen verwendet. Zur Abschätzung zeitlicher Effekte zwischen Kontrollzeitraum und Interventionszeitraum dienten die Datensätze der Vergleichskollektive der Cluster-II Modellklinik, des AKTIN-Notaufnahmeregister und des WIdO-Instituts. Die statistische Auswertung der Studiendaten erfolgte durch das evaluierende IGES-Institut (Berlin). p-Werte kleiner als 0.05 wurden als statistisch signifikant betrachtet. Alle statistischen Analysen wurden mit der Open Source Statistik Software R durchgeführt.

*A Posteriori Poweranalyse*

Zur Evaluation der Studienergebnisse wurde auf Basis der rekrutierten Fallzahlen in den Cluster-I Modellkliniken eine A Posteriori Poweranalyse durchgeführt, da die im Studienprotokoll avisierte Fallzahlplanung aufgrund der rückläufigen Patientenzahlen in der Pandemie und der nachträglichen Zuordnung von drei Modellkliniken in Cluster-II adjustiert werden musste.

Für die Analysen des angepassten primären Endpunktes lag mit insgesamt 58.702 validen Fällen in den acht Cluster-I Modellkliniken eine ausreichende A Posteriori-Power von 91 % vor: Diese A Posteriori Poweranalyse für den erwarteten Effekt von OPTINOFA (zweiseitiger Test, Alpha = 0,05) im Rahmen des Stepped-Wedge-Designs wurde unter denselben Annahmen wie die A Priori Poweranalyse, jedoch mit den tatsächlich realisierten Fallzahlen in acht Modellkliniken, durchgeführt (R-Pakets swCRTdesign): Binomialverteilung des Outcomes (0/1-Variable: 0 = stationär, 1 = ambulant) gemessen in acht Cluster-I Modellkliniken über 23 Monate im jeweiligen Kontroll- bzw. Interventionszeitraum. Die Analyse erfolgte auf der Grundlage eines verallgemeinerten, gemischten linearen Modells mit Random Effekten für Intercept und Zeitraumeffekt, womit zufällig auftretende Unterschiede in der Zuweisungsrate in die stationäre oder ambulante Behandlung in der Notaufnahme und der Umsetzung von OPTINOFA zwischen den Modellkliniken modelliert wurden. Hierbei wurde angenommen, dass die Zufallseffekte einer Normalverteilung mit Mittelwert Null und einer Standardabweichung von jeweils 0,15 folgen und dass der Random Intercept und der Random Zeitraumeffekt eine moderate Korrelation von 0,2 aufweisen. Bei einer erwarteten Steigerung der Verweise in die vertragsärztliche Versorgung von 0% auf 18% lag die A posteriori Power für die Überprüfung dieses Endpunkts daher bei 91 %.

*Statistische Analysen*

Die Strategie der Datenanalysen ist in der nachstehenden Tabelle zusammengefasst:

| **Modellkliniken** | **Analysemethode** |
| --- | --- |
| **Alle Cluster-I Modellkliniken**  (Interventionseffekt von Kontroll- zum Interventionszeitraum in allen Modellkliniken mit Einsatz von OPTINOFA) | - Mixed Models für den Interventionseffekt (Unterschied zwischen Kontroll- und Interventionszeitraum) mit Random Intercept und Random Slope zur Berücksichtigung der Heterogenität der Modellkliniken - logistisches oder lineares Regressionsmodell je nach Endpunkt bzw. Two-Part Modell für diagnostische Übereinstimmung (logistisches Modell) und diagnostische Effizienz (lineares Modell) |
| **Per Protocol Cluster-I Modellkliniken**  (Interventionseffekt Kontroll- zum Interventionszeitraum in Modellkliniken mit protokoll-gerechter Anwendung von OPTINOFA) | - Multiple Regressionsmodelle - logistisches oder lineares Regressionsmodell bzw. Two-Part Modell für diagnostische Übereinstimmung (logistisches Modell) und diagnostische Effizienz (lineares Modell) |
| **Cluster-II Modellklinik**  (allgemeine Zeitraumeffekte von Kontroll- zum Interventionszeitraum) | - Multiple Regressionsmodelle - logistisches oder lineares Regressionsmodell bzw. Two-Part Modell für diagnostische Übereinstimmung (logistisches Modell) und diagnostische Effizienz (lineares Modell) |

***Tab. 1 Analysestrategie in der multizentrischen Interventionsstudie im Projekt OPTINOFA***

Für die primären und sekundären Studienendpunkte wurden zunächst deskriptive statistische Analysen durchgeführt. Darüber hinaus wurden Generalized Linear Mixed Effects Models zur Analyse der Cluster-I Modellkliniken angewendet. Dem Design entsprechend wurden zwei Ebenen gebildet^[[2]](#footnote-2)^: Level 1 = einzelne Notaufnahmevorstellungen (Fallebene), Level 2 = Modellklinik der Notaufnahmevorstellung (Klinikebene). Die Heterogenität der Modellkliniken wurde im Mittelwert des Endpunkts (Random Intercept) und im Interventionseffekt (Effekt des Zeitraums, d. h. Veränderung von Kontroll- zu Interventionszeitraum; Random Slope) berücksichtigt. Zudem wurde eine Adjustierung für Merkmale der Notfallpatient:innen bzw. der Notaufnahmevorstellungen in Bezug auf Alter, Geschlecht, Behandlungsdauer des Leitsymptoms, stationäre Aufnahmewahrscheinlichkeit des Leitsymptoms, Triage-Stufe, geschlossene BD-Praxen zum Vorstellungszeitpunkt durchgeführt. Je nach Skalenniveau des Endpunkts wurden lineare und nicht-lineare Regressionsmodelle genutzt. Für die gemeinsame Analyse der diagnostischen Übereinstimmung und diagnostischen Effizienz wurden Two-Part Modelle mit logistischer Regression für die diagnostische Übereinstimmung und linearer Regression für die diagnostische Effizienz bei Vorliegen der diagnostischen Übereinstimmung gerechnet. Für den primären Endpunkt in der ursprünglichen Operationalisierung wurden ausschließlich die Schließzeiten der BD-Praxen kontrolliert, da die Veränderung im Patientenkollektiv den erwarteten Effekt bedingen sollte.

Um die so beobachtete Veränderung von Kontroll- zu Interventionszeitraum in den Cluster-I Modellkliniken um allgemeine (ggf. pandemiebedingte) zeitliche Trends zu kontrollieren, wurde je nach Skalenniveau des Endpunkts ein lineares bzw. nicht-lineares Regressionsmodell für die Cluster-II MK berechnet. Der für die Cluster-II Modellklinik ermittelte Zeitraumeffekt wurde dabei dem Cluster-I Effekt gegenübergestellt. Dadurch wurde abgebildet, inwiefern in den Cluster-I Modellklinken zusätzlich zu (ggf. pandemiebedingten) zeitlichen Trends in der Cluster-II Modellklinik eine Veränderung zu beobachten war, die dann auf den Einsatz von OPTINOFA zurückgeführt werden konnte. Der um Zeiteffekte bereinigte Interventionseffekt wurde durch den Unterschied in den prädizierten Werten im Kontrollzeitraum und Interventionszeitraum zwischen den Cluster-I Modellklinken und den Cluster-II Modellklinik berechnet. Hierfür wurden die mittleren Ausprägungen aller Kovariaten im Modell verwendet.

Das Ergebnis der Analysen wurde jeweils in einer grafischen Darstellung der zeitlichen Veränderung von Kontroll- zu Interventionszeitraum für die Cluster-I Modellkliniken, die Cluster-II Modellklinik und der um diese Zeiteffekte bereinigten Veränderung für die Cluster-I Modellkliniken aufgenommen.

Da die Anwendung des OPTINOFA Assistenzdienstes nicht in allen Cluster-I Modellkliniken stringent und damit protokollgerecht durchgeführt wurde, erfolgte jeweils eine separate Analyse der Daten in den sog. Per Protocol Cluster-I Kliniken, die OPTINOFA protokollgerecht angewendet haben. Die Analyse erfolgte je nach Endpunkt in linearen oder logistischen Regressionsmodellen, wobei die Heterogenität zwischen den beiden Modellkliniken durch die Interaktion aus Interventionseffekt (Unterschied zwischen Kontroll- und Interventionszeitraum) und Modellklinik berücksichtigt wurde.

Die Daten des AKTIN Notaufnahmeregisters und des WIdOs wurden deskriptiv ausgewertet und mit den beobachteten Ausprägungen und zeitlichen Trends in den Modellkliniken verglichen.

1. Abweichend vom Studienprotokoll haben die Universitätsklinik Aachen, das Klinikum Stuttgart und die Universitätsklinik Leipzig aufgrund von organisatorischen Hürden nicht an der Studie teilgenommen. Von den städtischen Kliniken München hat sich die München-Klinik Bogenhausen beteiligt. Zusätzlich konnten das Klinikum Wolfsburg, das Städtisches Klinikum Braunschweig, das Städtisches Klinikum Wolfenbüttel und das Evangelische Krankenhaus Göttingen-Weende für die Studie rekrutiert werden. Eine Verzerrung ist durch diese Abweichung vom Studienprotokoll nicht zu erwarten, da beide angedachten Stichproben an Modellkliniken Convenience-Samples sind (wie im Studienprotokoll vorgesehen). [↑](#footnote-ref-1)
2. Hussey MA; Hughes JP. Design and analysis of stepped wedge cluster randomized trials. Contemporary Clinical Trials, 2007: 28(2), 182–191 [↑](#footnote-ref-2)
