# Supplementary material for: Structured triage in the emergency department via intelligent assistant service OPTINOFA: Results of a multicenter, cluster-randomized and controlled interventional study in Germany
Source: Med Klin Intensivmed Notfmed. 2024 Dec 16;120(7):585–95. [Article in German] doi: 10.1007/s00063-024-01229-6 (PMC12504124; doi:10.1007/s00063-024-01229-6)
Supplement: Supplementary file 2 — Supplement 2 Weitere Ergebnisse [file 63_2024_1229_MOESM2_ESM.docx]

**Supplement 2 – Weitere Ergebnisse**

**Abb. 1 Korrelation zwischen der Disposition (tatsächliches Versorgungsziel) und der**

**Der OPTINOFA-Empfehlung**

**
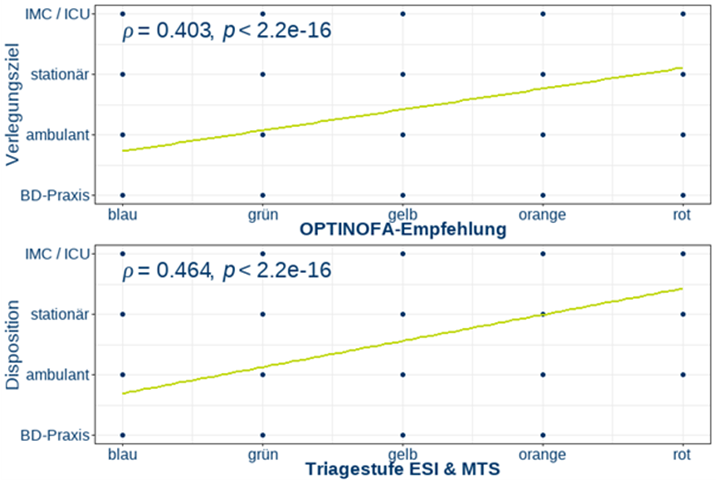
**

**Tab. 1 Primärer Endpunkt (angepasst)**

**Deskriptive Analyse der Zuweisungen zur BD-Praxis für Fälle mit den Dringlichkeitsstufen grün und blau (alle Cluster-I Modellkliniken)**

|  | **KZR (n = 13.596)** | | | | | **IZR (n = 11.451)** | | | |  | |
| --- | --- | --- | --- | --- | --- | --- | --- | --- | --- | --- | --- |
|  |  |  | | **95%-KI** | |  |  | **95%-KI** | |  |  |
| **Zuweisung** | **n** | **%** | | **LO** | **UP** | **n** | **%** | **LO** | **UP** | **Δ** | |
| *mit Einbezug der Zuweisungen an die KV (BD-Praxen)* | | | | | | | | | | | |
| NA stationär | 4.234 | | 31,14% | 30,37% | 31,93% | 3.514 | 30,69% | 29,85% | 31,54% | -0,45% | |
| NA ambulant | 8.195 | | 60,28% | 59,45% | 61,09% | 6.143 | 53,65% | 52,73% | 54,56% | -6,63% | |
| BD-Praxis | 1.167 | | 8,58% | 8,12% | 9,07% | 1.794 | 15,67% | 15,01% | 16,34% | 7,08% | |
| *ohne Einbezug der Zuweisungen an die KV (BD-Praxen)* | | | | | | | | | | | |
| NA stationär | 4.234 | | 34,07% | 33,24% | 34,90% | 3.514 | 36,39% | 35,43% | 37,35% | 2,32% | |
| NA ambulant | 8.195 | | 65,93% | 65,10% | 66,76% | 6.143 | 63,61% | 62,65% | 64,57% | -2,32% | |

Quelle: eCRF, Cluster-I; nur Notaufnahmevorstellungen mit den Triagestufen *grün* und *blau*

Anmerkung: KI = Konfidenzintervall; LO = Untere Grenze; UP = Obere Grenze; Berechnung von ∆ vor Rundung; KV = vertragsärztliche Versorgung

**Deskriptive Analyse der Zuweisungen zur BD-Praxis für Fälle mit den Dringlichkeitsstufen grün und blau (Per-Protocol Cluster-I Modellkliniken)**

|  | **KZR (n =2.034)** | | | | | **IZR (n = 2.043)** | | | |  | |
| --- | --- | --- | --- | --- | --- | --- | --- | --- | --- | --- | --- |
|  |  | |  | **95%-KI** | |  |  | **95%-KI** | |  |  |
| **Zuweisung** | **n** | | **%** | **LO** | **UP** | **n** | **%** | **LO** | **UP** | **Δ** | |
| *mit Einbezug der Zuweisungen an die KV (BD-Praxen)* | | | | | | | | | | | |
| NA stationär | 189 | 9,29% | | 8,11% | 10,63% | 48 | 2,35% | 1,78% | 3,10% | -6,94% | |
| NA ambulant | 865 | 42,53% | | 40,39% | 44,69% | 398 | 19,48% | 17,82% | 21,26% | -23,05% | |
| BD-Praxis | 980 | 48,18% | | 46,01% | 50,35% | 1.597 | 78,17% | 76,33% | 79,91% | 29,99% | |
| *ohne Einbezug der Zuweisungen an die KV (BD-Praxen)* | | | | | | | | | | | |
| NA stationär | 189 | 17,93% | | 15,73% | 20,36% | 48 | 10,76% | 8,21% | 13,98% | -7,17% | |
| NA ambulant | 865 | 82,07% | | 79,64% | 84,27% | 398 | 89,24% | 86,02% | 91,79% | 7,17% | |

Quelle: eCRF, Cluster-I (2 Per-Protocol MK); nur Notaufnahmevorstellungen mit den Triagestufen *grün* und *blau*

Anmerkung: KI = Konfidenzintervall; LO = Untere Grenze; UP = Obere Grenze; Berechnung von ∆ vor Rundung; KV = vertragsärztliche Versorgung

**Tab. 2 Primärer Endpunkt (original)**

**Deskriptive Analyse der ambulanten und stationären Zuweisungen für Fälle mit Behandlung in der Notaufnahme**

|  | **KZR (n = 7.992)** | | | | | **IZR (n = 5.253)** | | | |  | |
| --- | --- | --- | --- | --- | --- | --- | --- | --- | --- | --- | --- |
|  |  | |  | **95%-KI** | |  |  | **95%-KI** | |  |  |
| **Zuweisung** | **n** | | **%** | **LO** | **UP** | **n** | **%** | **LO** | **UP** | **Δ** | |
| *Cluster-I Modellkliniken* | | | | | | | | | | | |
| NA stationär | 16.994 | 54,48% | | 53,93% | 55,03% | 13.209 | 53,85% | 53,22% | 54,47% | -0,64% | |
| NA ambulant | 14.198 | 45,52% | | 44,97% | 46,07% | 11.322 | 46,15% | 45,53% | 46,78% | 0,64% | |
| *Cluster-I Per-Protocol Modellkliniken* | | | | | | | | | | | |
| NA stationär | 4.036 | 57,56% | | 56,40% | 58,71% | 1.955 | 53,47% | 51,85% | 55,09% | -4,08% | |
| NA ambulant | 2.976 | 42,44% | | 41,29% | 43,60% | 1.701 | 46,53% | 44,91% | 48,15% | 4,08% | |
| *Cluster-II Modellklinik* | | | | | | | | | | | |
| NA stationär | 1.616 | 41,76% | | 40,21% | 43,32% | 1.286 | 40,79% | 39,08% | 42,51% | -0,97% | |
| NA ambulant | 2.254 | 58,24% | | 56,68% | 59,79% | 1.867 | 59,21% | 57,49% | 60,92% | 0,97% | |

Quelle: eCRF, nur Behandlungen in der Notaufnahme

Anmerkung: KI = Konfidenzintervall; LO = Untere Grenze; UP = Obere Grenze; Berechnung von ∆ vor Rundung; KV = vertragsärztliche Versorgung

**Abb. 2 Inferenzstatistik primärer Endpunkt (original)**

**Geringe Veränderung in der Zuweisung zu ambulanter bzw. stationärer Versorgung in der Notaufnahme im Interventionszeitraum im Vergleich zum Kontrollzeitraum in den Cluster-I Kliniken**

~~
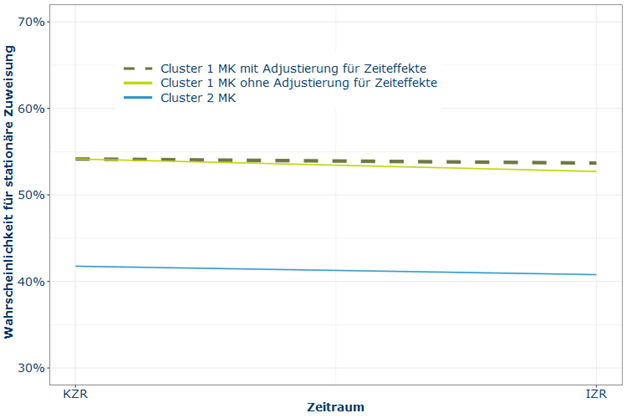
~~

Alle Cluster-I Modellkliniken

**Tab. 3 Sekundärer Endpunkt:**

**Outcome - Anteil stationärer Aufnahmen von ambulanten Notfallbehandlungen innerhalb**

**von 3 Tagen**

|  | **KZR** | | | | **IZR** | | | |  |
| --- | --- | --- | --- | --- | --- | --- | --- | --- | --- |
| **Tag nach Vorstellung** | **n stat** | **%** | **LO** | **UP** | **n stat** | **%** | **LO** | **UP** | **∆** |
| *Alle Cluster-I MK* | *(n=5.946)* | | | | *(n=4.949)* | | | |  |
| 0-3 Tage | 264 | 4,44% | 3,95% | 4,99% | 239 | 4,83% | 4,27% | 5,46% | 0,39% |
| *Per Protocol MK* | *(n=2.181)* | | | | *(n=1.611)* | | | |  |
| 0-3 Tage | 127 | 5,82% | 4,92% | 6,89% | 108 | 6,70% | 5,58% | 8,03% | 0,88 |
| *Cluster-II MK* | *(n=723)* | | | | *(n=632)* | | | |  |
| 0-3 Tage | 25 | 3,46% | 2,35% | 5,05% | 30 | 4,75 | 3,34% | 6,70% | 1,29% |

Quelle: eCRF, Routinedaten der Krankenkassen; nur ambulante Zuweisungen (NA ambulant, BD-Praxis)

Anmerkung: n stat = Fälle mit stationärer Aufnahme; LO = Untere Grenze 95 %-KI; UP = Obere Grenze 95 %-KI; Berechnung von ∆ vor Rundung

**Tab. 4**  **Sekundärer Endpunkt:**

**Wartezeit in Minuten bis zum ersten Pflege- bzw. Arztkontakt**

|  | **KZR** | | | | **IZR** | | | |  |  |
| --- | --- | --- | --- | --- | --- | --- | --- | --- | --- | --- |
| **Wartezeit** | **n** | **M** | **SD** | **Md** | **n** | **M** | **SD** | **Md** | **ΔM** | **ΔM (%)** |
| *Alle Cluster-I MK* | | | | |  |  |  |  |  |  |
| Arzt | 30.608 | 59,20 | 58,45 | 38 | 24.247 | 48,36 | 49,76 | 31 | -10,85 | -18,32% |
| Pflege | 28.135 | 3,65 | 4,98 | 2 | 23.846 | 3,51 | 5,25 | 1 | -0,15 | -4,06% |
| *Per Protocol MK* | | | | |  |  |  |  |  |  |
| Arzt | 6.759 | 69,44 | 67,04 | 45 | 3.559 | 49,62 | 61,25 | 23 | -19,82 | -28,54% |
| Pflege | 6.813 | 6,27 | 5,17 | 5 | 3.550 | 5,01 | 4,70 | 4 | -1,26 | -20,16% |
| *Cluster-II MK* | | | | |  |  |  |  |  |  |
| Arzt | 3.803 | 47,39 | 54,24 | 26 | 3.110 | 42,76 | 53,65 | 20 | -4,62 | -9,75% |
| Pflege | 2.744 | 7,71 | 6,75 | 6 | 2.385 | 7,88 | 6,47 | 6 | 0,17 | 2,16% |

Quelle: eCRF, , nur Behandlungen in der Notaufnahme (NA stationär, NA ambulant)

Anmerkung: ΔM = Mittelwertsdifferenz; Berechnung von ∆M vor Rundung

**Tab. 5 Sekundärer Endpunkt:**

**Verweildauer in der Notaufnahme in Minuten**

| **Gruppe** | **KZR** | | | | **IZR** | | | |  | |
| --- | --- | --- | --- | --- | --- | --- | --- | --- | --- | --- |
|  | **n** | **M** | **SD** | **Md** | **n** | **M** | **SD** | **Md** | **ΔM** | **ΔM (%)** |
| Alle Cluster-I MK | 16.758 | 281,21 | 163,13 | 254 | 13.074 | 271,43 | 147,88 | 243 | -9,78 | -3,48% |
| Cluster-II MK | 1.615 | 252,08 | 156,01 | 221 | 1.285 | 287,74 | 170,51 | 262 | 35,66 | 14,15% |
| Per-Protocol MK | 3.965 | 321,66 | 196,70 | 292 | 1.849 | 363,36 | 235,79 | 316 | 41,69 | 12,96% |
| AKTIN-Kliniken | 30.108 | 218,30 | 124,76 | 191 | 24.808 | 224,80 | 118,59 | 202 | 6,51 | 2,98% |

Quelle: eCRF, Cluster-I (insgesamt und Per-Protocol MK separat), Cluster-II MK, nur stationäre Zuweisungen (NA stationär), AKTIN-Daten

Anmerkung: ΔM = Mittelwertsdifferenz; Berechnung von ∆M vor Rundung


**Tab. 6 Sekundärer Endpunkt:**

**Mittlere Kosten der Notfallbehandlung, Per-Protocol Modellkliniken**

|  | **KZR (n=4.658)** | | **IZR (n=2.760)** | |  |  |
| --- | --- | --- | --- | --- | --- | --- |
| **Kostenquelle** | **M** | **SD** | **M** | **SD** | **∆M** | **∆M (%)** |
| stationär | 2.852 € | 3.662 € | 2.572 € | 3.774 € | -279 € | -9,80% |
| AMBO | 13 € | 69 € | 17 € | 98 € | 3 € | 24,22% |
| Ambulant (EFN) | 152 € | 399 € | 155 € | 406 € | 3 € | 1,96% |
| gesamt | 3.017 € | 3.666 € | 2.744 € | 3.782 € | -273 € | -9,06% |
| n Kontakte | 2,61 | 1,65 | 2,62 | 1,70 | 0,01 | 0,38% |

Quelle: eCRF, Routinedaten der Krankenkassen, Cluster-I (2 Per-Protocol MK)

Anmerkung: ∆M = Mittelwertdifferenz; Berechnung von ∆M vor Rundung; EFN = Einzelfallnachweise; n Kontakte = mittlere Anzahl Behandlungskontakte innerhalb von 28 Tagen
